# Family Functioning and Adolescent Depression: Parallel and Serial Mediation Roles of Academic Stress and Emotion Regulation

**DOI:** 10.3390/bs16020244

**Published:** 2026-02-09

**Authors:** Mingping Jiang, Haibo Yang

**Affiliations:** 1 School of Education and Psychological Science, Hefei Normal University, Hefei 230061, China; jiangmingping@hfnu.edu.cn; 2 Key Laboratory of Philosophy and Social Sciences of Anhui Province on Adolescent Mental Health and Crisis Intelligent Intervention, Hefei Normal University, Hefei 230061, China; 3 Academy of Psychology and Behavior, Faculty of Psychology, Tianjin Normal University, Tianjin 300387, China

**Keywords:** family functioning, depression, academic stress, emotion regulation strategies, adolescents

## Abstract

With the rapid pace of economic development and intensifying social competition, adolescent depression has emerged as an escalating global public health concern. The present study investigated the relationship between family functioning and adolescent depression, with particular attention being paid to the parallel and serial mediating roles of academic stress and emotion regulation strategies. A total of 437 adolescents from Anhui Province were surveyed using the Chinese versions of the Family Assessment Device, the Academic Stress Scale, the Emotion Regulation Questionnaire, and the Center for Epidemiologic Studies Depression Scale (CES-D). The results revealed that (1) the prevalence of depression was 27.7%, with 31.2% of participants experiencing moderate to high levels of academic stress; (2) family functioning was identified as a key predictor of adolescent depression; and (3) academic stress and expressive suppression sequentially mediated the relationship between family functioning and depression, while academic stress and cognitive reappraisal functioned as parallel mediators. In conclusion, healthy family functioning plays a crucial role in reducing adolescent depression, both directly and through the mediating effects of academic stress and emotion regulation strategies. These findings highlight the importance of family support and the adoption of adaptive coping mechanisms in promoting adolescent mental health.

## 1. Introduction

Adolescent depression has become a pressing global public health concern in recent years. Epidemiological data suggest that approximately 24.0% of adolescents exhibit depressive symptoms, with over 7.0% meeting the criteria for moderate to severe depression—a prevalence that has risen significantly since the pre-COVID-19 period (Fu & Zhang, 2021). It is now recognized as one of the leading causes of disability worldwide (De Sousa et al., 2025). Adolescents are particularly vulnerable because of their developing emotion regulation and cognitive capacities, rendering them more susceptible to significant psychological disorders and increasing their risk for depression later in life (Lim & Kwon, 2023).

Extensive research has demonstrated that academic stress is a major contributor to psychological distress in adolescence. Factors such as exam-oriented education systems, excessive academic workloads, intense competition, and high parental expectations collectively undermine adolescent mental health (N. Xu et al., 2019). At the same time, family functioning plays a pivotal role in the development of depressive symptoms. When family dynamics are impaired and psychological resilience is diminished, negative emotions tend to accumulate, thereby compromising adolescents’ psychological and physical well-being (J. Zhang et al., 2024). According to Ecological Systems Theory, the family, as a core microsystem, affects adolescent health through multiple pathways. A supportive family environment and effective parental involvement serve as buffers against stress and help mitigate the risk of depression (S. Chen et al., 2024).

Additionally, emotion regulation strategies are closely linked to depression: the effective use of such strategies can substantially decrease risk. However, shaped by cultural norms that value emotional restraint, Chinese adolescents often rely on expressive suppression—a strategy that may perpetuate maladaptive rumination and significantly increase vulnerability to depression (J. Wang, 2018). Thus, a systematic examination of these interconnected factors is essential for the development of effective early intervention and prevention programs in behavioral science.

### 1.1. The Relationship Between Family Functioning and Depression

Systematic reviews and meta-analyses have demonstrated a significant association between family dysfunction and adolescent depression (Daniel et al., 2020). Specifically, higher levels of depressive symptoms in adolescents are closely correlated with perceived parental rejection and impaired family functioning (Sünbükel & Şahin, 2016). In contrast, effective family functioning not only cultivates gratitude among both parents and adolescents but also reduces depressive symptoms, thereby promoting positive emotions and enhanced psychological well-being in both groups (Yeung, 2025). Moreover, effective family functioning can buffer the negative effects of sleep disturbances on depressive symptoms among primary and secondary school students (Wen et al., 2023) and may alleviate depression and social anxiety in adolescents by strengthening their belief in a just world (J. Tang et al., 2025).

Longitudinal research has further shown that family functioning moderates the relationship between depression treatment and suicidal ideation; among adolescents from well-functioning families, interventions significantly reduce suicidal thoughts (Dardas, 2019). The impact of family functioning appears particularly salient in vulnerable populations. For adolescents with chronic illnesses, poor functioning of primary caregivers directly elevates the risk of depression and reduces quality of life (Zhu et al., 2023). Ferro and Boyle (2015) reported that children with chronic physical illnesses often experience higher levels of anxiety and depression, frequently accompanied by increased maternal depression, severe family dysfunction, and diminished self-esteem. Similarly, Yeh et al. (2016) found that family functioning serves as a mediator for the relationship between maternal depression and both positive and negative affect in adolescents, suggesting that strong family functioning may help buffer against the intergenerational transmission of depressive symptoms. Notably, adolescents and their parents often differ in their perceptions of family functioning and parent–child relationships. These differences can manifest as unclear role boundaries, imbalanced emotional involvement, and the neglect of inner emotional experiences (Q. Chen et al., 2017).

In light of this evidence, the present study aims to further investigate the relationship between family functioning and adolescent depression, and accordingly proposes Research Hypothesis 1 (H1): Family functioning is a significant predictor of adolescent depression.

### 1.2. The Mediating Role of Academic Stress

While the direct impact of family functioning on depression is well established, the specific mediating mechanisms underlying this relationship remain not fully understood. Existing literature often addresses individual mediators—for example, the role of psychological resilience in linking only-child status to depression (M. Zhang & Gao, 2023)—or broadly examines indirect effects, such as family functioning mediating the association between daily activities and depressive symptoms (Yang et al., 2020). However, there is a notable lack of systematic investigation into intrinsic stressors within the family environment, particularly academic stress, which is central to family dynamics.

Academic stress is defined as the psychological burden and tension arising from various educational sources, including pressure from parents, oneself, teachers, and peers (J. Xu et al., 2010). Longitudinal research with Chinese secondary school students has identified a moderate positive correlation between academic stress and depressive symptoms; moreover, the level of academic stress significantly predicts the severity of depression (L. Wang & Jiang, 2022). Similar associations have been reported in other populations, such as first-year nursing students (Cho et al., 2015). Importantly, this relationship appears to vary by gender: while male depressive symptoms are more influenced by peer relations, academic stress is more strongly linked to depression in females (Yun & Lee, 2018). Additional studies suggest that factors such as smartphone addiction can sequentially moderate the relationship between academic stress and depression (X. Zhang et al., 2022), and that perfectionism influences depression through the mediating effect of academic stress (Zhou et al., 2024). Collectively, these findings highlight academic stress as not only an independent risk factor for depression but also a key pathway linking more distal risk factors to immediate psychopathology.

Academic stress thus serves as a vital bridge between the family system and adolescent depression. In well-functioning families, emotional support, clear structure, and a problem-solving orientation help youth confront academic challenges and reduce self-criticism, thereby lowering the risk for internalizing symptoms. By contrast, dysfunctional family environments may exacerbate internalized stress through rigid parenting practices (Y. Zhang et al., 2021). Within collectivist cultures, academic pressure from fathers tends to be particularly likely to contribute to depressive and anxious symptoms—potentially reflecting traditional family roles and authoritarian parenting (Quach et al., 2015). This suggests that family functioning directly influences both the level and nature of academic stress. Additionally, Wei et al. (2025) found that younger adolescents are especially vulnerable to academic stress due to poor sleep and lower psychological resilience, indicating that family functioning may both contribute to the generation of stress and shape coping resources, such as resilience, thereby moderating the effects of stress.

Although prior research has independently established links between academic stress and depression, as well as between family functioning and academic stress, few studies have explored the interplay among these variables. Therefore, the current study seeks to elucidate the mediating role of academic stress in the relationship between family functioning and adolescent depression.

### 1.3. The Mediating Role of Emotion Regulation Strategies

Gross (1998, 2015) argued that cognitive reappraisal—an adaptive, antecedent-focused emotion regulation strategy that involves changing how one interprets emotional events—is inversely related to depression, whereas expressive suppression—a maladaptive, response-focused strategy involving the inhibition of emotional expression—is positively associated with depression. Kraft et al. (2023), in a meta-analysis of 181 studies, further found that maladaptive strategies (e.g., rumination, avoidance, suppression) exert a greater overall effect on internalizing symptoms and are stronger predictors of depression than adaptive strategies. This line of research underscores that emotion regulation strategies are proximal, modifiable predictors of depression, with empirical evidence supporting both their causal influence and contextual sensitivity. For example, Sullivan et al. (2023) demonstrated that sleep quality impacts depression through specific emotion regulation patterns; similarly, Łubińska Salej and Radziwiłłowicz (2025) reported that reduced mentalizing capacity predicts depression severity via maladaptive regulation strategies. Notably, recent findings by Xiao et al. (2025) revealed that the impact of cognitive reappraisal is highly context-dependent: while it may alleviate depression in low-constraint environments, it can actually increase anxiety and depression in highly restrictive, high-stress family settings.

Research has also documented a close relationship between family functioning and emotion regulation strategies. For example, Su et al. (2024) reported that maternal expressive suppression predicts adolescent depressive symptoms and moderates the relationship between adolescent suppression and depression, highlighting the likelihood of intergenerational transmission of emotion regulation habits. These results indicate that specific parental emotion regulation styles can be internalized by adolescents as personal coping strategies. Yin et al. (2022) further supported this by linking childhood trauma with increased expressive suppression, with emotion regulation strategies both mediating and moderating this relationship. Since childhood trauma often stems from poor family functioning (e.g., abuse, neglect), dysfunctional family dynamics can intensify traumatic stress, thereby fostering maladaptive strategies such as suppression and consequently increasing the risk for depression. Cross-cultural research by Martínez-Líbano et al. (2025) also confirms these findings, showing that emotional clarity—encouraged through emotional affirmation and feedback in family settings—significantly moderates the relationship between emotion regulation and depression.

In summary, while previous research has established associations between emotion regulation strategies and depression, as well as between family functioning and emotion regulation, few studies have clarified the mechanisms underlying these connections. Accordingly, this study aims to examine whether cognitive reappraisal and expressive suppression mediate the association between family functioning and depression.

### 1.4. Parallel and Sequential Mediating Roles of Academic Stress and Emotion Regulation Strategies

Previous research has primarily examined family functioning, academic stress, emotion regulation strategies, and depression as separate factors. Although it is well established that academic stress can undermine adolescent development—leading to poorer academic performance, reduced self-esteem, and higher rates of depression and anxiety—and that family dysfunction is a significant risk factor for depression, the specific pathways connecting these variables remain unclear. Key questions persist: Through which mechanisms does academic stress lead to depressive symptoms? Does family functioning mitigate or exacerbate this process? And what role do emotion regulation strategies play? Addressing these gaps requires an integrated framework to systematically explore the dynamic interplay among these variables.

Ecological systems theory conceptualizes development as ongoing interactions between individuals and their layered environments, with the family microsystem critically shaping adolescents’ coping resources (J. Liu & Meng, 2009). Emotion regulation theory suggests that individuals employ specific strategies—such as cognitive reappraisal (antecedent-focused) or expressive suppression (response-focused)—to manage emotional responses, with contextual factors influencing strategy selection. Furthermore, the stress-coping model proposes that individuals appraise stressors, select coping strategies, and thereby shape psychological outcomes (Lazarus & Folkman, 1984).

By integrating these perspectives, it is plausible that academic stress and emotion regulation strategies mediate the relationship between family functioning and depression, with academic stress likely preceding emotion regulation in the causal process. Moreover, these factors may act in parallel or sequentially. To address these mechanisms, we propose the following hypotheses:

**Hypothesis** **1** **(H1).** *Family functioning is a significant predictor of adolescent depression.*

**Hypothesis** **2** **(H2).** *Academic stress and emotion regulation strategies (cognitive reappraisal and expressive suppression) act as parallel mediators between family functioning and depression.*

**Hypothesis** **3** **(H3).** *Academic stress and emotion regulation strategies (cognitive reappraisal and expressive suppression) operate as sequential (chain) mediators between family functioning and depression.*

**Figure 1:**
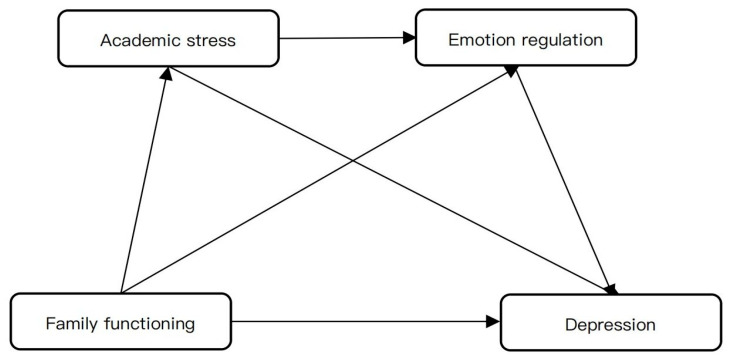
Theoretical model diagram.

This study expands on previous research by simultaneously evaluating both parallel and sequential mediation models, providing a more nuanced understanding of how academic stress and emotion regulation contribute to adolescent depression. Our aim is to clarify the dynamic pathways linking family functioning to individual psychological processes in adolescents. A visual representation of the proposed model can be found in Figure 1.

## 2. Materials and Methods

### 2.1. Research Instruments

#### 2.1.1. Chinese Version of the Family Assessment Device

The Chinese Revised Edition of the Family Assessment Device (FAD) was utilized to evaluate adolescents’ perceptions of family functioning. The scale was originally developed by Epstein et al. based on the McMaster Model of Family Functioning; its cross-cultural adaptation required local validation. R. Li et al. (2013) adapted and validated the instrument for a normative Chinese sample, resulting in a 30-item measure with five dimensions: emotional communication, positive communication, egocentricity, problem-solving, and family rules. Each item is rated on a 4-point Likert scale, and total scores are calculated as the mean across items; higher scores correspond to better perceived family functioning.

The Chinese version of the FAD demonstrates robust psychometric properties in adolescent samples. Confirmatory factor analysis supported the five-factor solution, indicating good model fit (χ^2^/df = 2.99, RMSEA = 0.05, NNFI = 0.95, CFI = 0.95). The internal consistency coefficient for the total scale was high (α = 0.91), while the subscales yielded Cronbach’s α coefficients ranging from 0.60 to 0.85. Previous studies reported a Cronbach’s α of 0.82 for the full scale, which is consistent with the α of 0.81 found in the current study.

#### 2.1.2. Academic Stress Scale

Academic stress was assessed using the Academic Stress Scale developed by J. Xu et al. (2010), a 21-item instrument that covers four domains: parental pressure, self-pressure, teacher pressure, and social pressure, rated on a 5-point Likert scale. Aggregate scores span from 21 to 105, where higher scores indicate greater academic stress. Low stress (21–49) reflects normal adaptation and manageable stress levels; moderate stress (50–68) implies a need for ongoing monitoring and psychological support; and high stress (69–105) marks significant psychological risk that may necessitate professional intervention. Subsequent research has consistently demonstrated good reliability (test–retest coefficients from 0.80 to 0.86) and strong structural validity for the Chinese version. Subscale-to-total correlations ranged from 0.59 to 0.76, indicative of satisfactory convergent validity. In this study, the Cronbach’s α was 0.89.

#### 2.1.3. Chinese Version of the Emotion Regulation Questionnaire

Cognitive emotion regulation was measured using the Chinese version of the Emotion Regulation Questionnaire (ERQ), revised by L. Wang et al. (2007). This 10-item scale assesses two primary dimensions: cognitive reappraisal (an antecedent-focused strategy that changes the emotional impact of events through reinterpretation) and expressive suppression (a response-focused strategy aimed at inhibiting ongoing emotional expression). Respondents rate items on a 7-point Likert scale. Initial validation of the Chinese ERQ found test–retest reliability coefficients of 0.82 and 0.79, with Cronbach’s α values of 0.85 for the reappraisal subscale and 0.77 for the suppression subscale. Confirmatory factor analysis confirmed the two-factor model, yielding acceptable fit indices (χ^2^/df = 9.40, IFI = 0.96, CFI = 0.96, NFI = 0.95, NNFI = 0.95, RMSEA = 0.085). In the present sample, Cronbach’s α values were 0.85 and 0.81, respectively.

#### 2.1.4. Chinese Short Version of the Center for Epidemiological Studies Depression Scale

Depressive symptoms were assessed with the Chinese Short Version of the Center for Epidemiological Studies Depression Scale (CES-D). Initially developed by Radloff and later adapted for Chinese samples by Z. Chen et al. (2009), this 20-item measure encompasses four domains: depressive mood, positive affect, somatic symptoms, and interpersonal difficulties. Items 4, 8, 12, and 16 are reverse-coded. Each item is rated on a 4-point Likert scale (0–3). The Chinese CES-D has demonstrated high internal consistency (α = 0.83), with Cronbach’s α ranging from 0.77 to 0.88 in community samples and from 0.85 to 0.90 in clinical groups. The scale’s structure also shows excellent model fit, as evidenced by the following indices: χ^2^ = 7601.97, df = 164, RMSEA = 0.067, NFI = 0.958, NNFI = 0.953, CFI = 0.959, IFI = 0.959, GFI = 0.931, AGFI = 0.911. In this study, Cronbach’s α was 0.91.

### 2.2. Participants and Procedure

Informed consent was actively obtained from schools, parents, and students prior to data collection. Online testing was conducted in accordance with a standardized protocol: (1) All research administrators and homeroom teachers completed standardized training; (2) The survey was delivered via www.wjx.cn on 10 June 2025, with embedded attention-check items to ensure data quality; (3) Real-time monitoring was implemented to identify anomalous response patterns (e.g., straight-lining or zigzag responses) for immediate follow-up.

All measures were administered during a single session. Given the cross-sectional design, this study can detect associations among variables but cannot determine causality or assess changes over time. Nevertheless, this methodology facilitated efficient data collection and supported the use of structural equation modeling for analysis.

Participants were selected using stratified cluster random sampling. A total of 460 students were randomly chosen from three grade levels at a public junior high school in Anhui Province, China. Exclusion criteria included: (1) Missing data on more than 5% of items, or omission of three or more items within any single scale; (2) Failure on two or more attention-check items; (3) Ten or more consecutive identical responses, or conspicuously patterned (e.g., alternating) responses.

After data screening, 437 valid questionnaires remained (valid response rate = 95.0%). The final sample comprised 232 boys (53.1%) and 205 girls (46.9%), distributed by grade as follows: 163 seventh-graders (37.3%), 154 eighth-graders (35.2%), and 120 ninth-graders (27.5%). Additionally, 146 participants (33.4%) were only children, and 291 (66.6%) had siblings. All analyses were performed using this finalized dataset.

### 2.3. Statistical Analysis

Data were collected through an online survey platform (www.wjx.cn) on 10 June 2025. Prior to participation, electronic informed consent was obtained from both students and their parents, and the study protocol was approved by the institutional ethics committee.

All statistical analyses were carried out using SPSS 26.0 and the PROCESS macro (Model 4 for simple mediation and Model 6 for serial mediation). Descriptive statistics, independent-samples *t*-tests, and analyses of variance (ANOVA) were conducted to examine group differences. Normality was assessed using the Shapiro–Wilk test and Q–Q plots prior to hypothesis testing, and homogeneity of variance was checked using Levene’s test; when necessary, Welch’s correction was applied to address violations. For mediation analyses, bias-corrected bootstrapping with 5000 resamples was employed to generate 95% confidence intervals for indirect effects; intervals not containing zero were considered statistically significant. Diagnostic checks confirmed that all statistical assumptions were met, supporting the reliability of the analyses. Statistical significance was set at *p* < 0.05 (two-tailed).

## 3. Results

### 3.1. Common Method Bias Test

Given this study’s exclusive reliance on self-report questionnaires, the potential for common method bias was carefully examined. To assess this, Harman’s single-factor test was applied to all questionnaire items prior to further analyses. Specifically, an exploratory factor analysis was conducted, with the extraction constrained to a single unrotated factor using either principal component analysis or maximum likelihood estimation. Common method bias was considered present if this single factor explained more than 40% of the total variance, in accordance with common empirical guidelines. The analysis identified 17 factors with eigenvalues greater than 1, and the first factor accounted for only 27.56% of the total variance—well below the 40% threshold. Thus, common method bias is unlikely to pose a significant concern in this study.

### 3.2. Overall Status of Adolescent Depression and Academic Stress

As previously described, higher academic stress scores reflect greater perceived stress, with score ranges defined as follows: 21–49 (low stress), 50–68 (moderate stress), and 69–105 (high stress). In this sample, 301 students (68.9%) were classified as experiencing low stress, 113 (25.9%) as moderate stress, and 23 (5.3%) as high stress.

For depression, following Radloff’s classification system (as adapted by Z. Chen et al., 2009), CES-D scores of 1–15 indicate no depression, 16–27 indicate mild depression, and scores of 28 or above indicate high depression. Based on these thresholds, 84 participants (19.2%) exhibited mild depressive symptoms, while 37 (8.5%) were classified as experiencing high levels of depression. Furthermore, the prevalence of depressive symptoms was significantly higher among students in higher grades compared to those in lower grades (*p* < 0.05).

### 3.3. Differences Across Demographic Variables

The influence of demographic variables on perceived family functioning, academic stress, and depression was analyzed. Results revealed significant gender differences in both family functioning (t = 2.49, *p* < 0.05) and cognitive reappraisal (t = 4.96, *p* < 0.001), with males scoring higher than females. Conversely, females reported significantly higher academic stress (t = −1.97, *p* < 0.05) than males. Significant grade-level differences were also observed for family functioning (F = 7.83, *p* < 0.001), academic stress (F = 5.79, *p* < 0.001), expressive suppression (F = 14.25, *p* < 0.001), and depression (F = 7.71, *p* < 0.001). Specifically, seventh-grade (first-year) students demonstrated significantly higher family functioning scores compared to eighth- and ninth-grade (second- and third-year) students. In terms of academic stress and expressive suppression, students in eighth and ninth grades reported significantly higher scores than those in seventh grade. Similarly, depression scores were significantly higher among eighth- and ninth-graders compared to seventh-graders. All observed differences were statistically significant (see Table 1).

### 3.4. Correlation Matrix of Family Functioning, Academic Stress, Emotion Regulation Strategies, and Depression in Adolescents

Correlation analyses revealed significant relationships among family functioning, academic stress, the emotion regulation strategies of cognitive reappraisal and expressive suppression, and depression in adolescents. Family functioning was significantly negatively correlated with academic stress, expressive suppression, and depression, while showing a significant positive correlation with cognitive reappraisal. Both academic stress and expressive suppression were significantly positively correlated with depression; additionally, academic stress was significantly negatively correlated with cognitive reappraisal. Cognitive reappraisal was also significantly negatively correlated with depression, whereas expressive suppression was significantly positively correlated with depression (*p* < 0.01; see Table 2).

### 3.5. Mediating Effects of Academic Stress and Emotion Regulation Strategies

Given the significant associations among family functioning, academic stress, emotion regulation strategies, and depression, further analyses were conducted to examine the mediating roles of academic stress and emotion regulation strategies. Mediation analyses using bootstrapping procedures were performed with Hayes’s SPSS PROCESS macro (Model 6). Results indicated that the mediating effects of the two emotion regulation strategies—cognitive reappraisal and expressive suppression—differed significantly and were therefore analyzed separately.

After controlling for gender and grade, family functioning was entered as the independent variable, adolescents’ depressive tendency as the dependent variable, and academic stress, along with expressive suppression as mediators.

Regression analyses revealed that family functioning had a significant direct effect on adolescent depression (direct effect = 0.3584, accounting for 56.35% of the total effect). Critically, the direct effect reflects the unique contribution of family functioning to depression after accounting for the mediators, and should not be conflated with model fit indices such as R^2^. Family functioning significantly and negatively predicted academic stress (β = −0.8376, *p* < 0.001) and expressive suppression (β = −0.9104, *p* < 0.001). Academic stress, in turn, significantly and positively predicted expressive suppression (β = 0.2932, *p* < 0.001) and depression (β = −0.0764, *p* < 0.001).

Mediation analysis indicated that academic stress and expressive suppression contributed significant indirect effects in the relationship between family functioning and adolescent depression (indirect effect = −0.2776, accounting for 43.65% of the total effect). The total indirect effect consisted of three specific pathways: (1) family functioning → academic stress → depression (indirect effect 1 = −0.1892); (2) family functioning → expressive suppression → depression (indirect effect 2 = −0.0696); and (3) family functioning → academic stress → expressive suppression → depression (indirect effect 3 = −0.0188). These pathways accounted for 29.70%, 10.94%, and 2.96% of the total effect, respectively. Importantly, the 95% confidence intervals for all three indirect effects did not include zero, indicating statistical significance.

**Figure 2:**
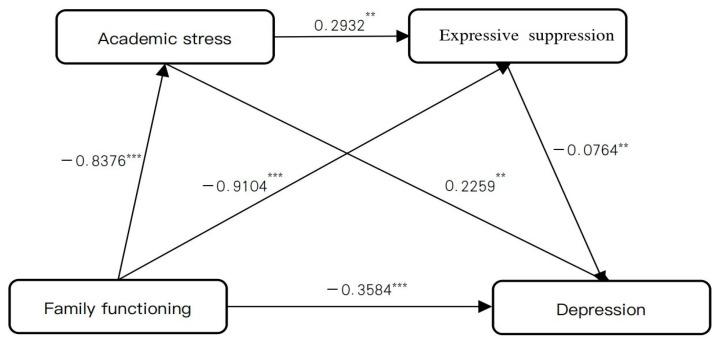
The mediating path diagram of academic stress and expressive suppression. **Note:** *** *p* < 0.001, ** *p* < 0.01.

Overall, these findings demonstrate that academic stress and expressive suppression serve as chain mediators in the relationship between family functioning and adolescents’ depressive tendencies, forming the pathway: family functioning → academic stress → expressive suppression → depression (see Table 3 and Table 4, and Figure 2).

Similarly, after controlling for gender and grade, the mediating roles of academic stress and cognitive reappraisal were examined, with family functioning specified as the independent variable and adolescents’ depressive tendency as the dependent variable. Regression results showed that family functioning had a significant direct effect on adolescent depression (direct effect = 0.3694, accounting for 58.10% of the total effect). Specifically, family functioning positively predicted cognitive reappraisal (β = 0.7839, *p* < 0.001) and negatively predicted academic stress (β = −0.8376, *p* < 0.001). In turn, academic stress positively predicted adolescents’ depression (β = 0.2324, *p* < 0.001).

Mediation analysis revealed that both academic stress and cognitive reappraisal were significant mediators in the relationship between family functioning and adolescent depression (indirect effect = 0.2665, accounting for 41.90% of the total effect). The total indirect effect comprised two distinct pathways: (1) family functioning → academic stress → depression (indirect effect 1 = −0.1947); and (2) family functioning → cognitive reappraisal → depression (indirect effect 2 = −0.0718). These two indirect effects accounted for 30.61% and 11.29% of the total effect, respectively. Notably, the 95% confidence intervals for both indirect effects did not include zero, indicating statistical significance.

**Figure 3:**
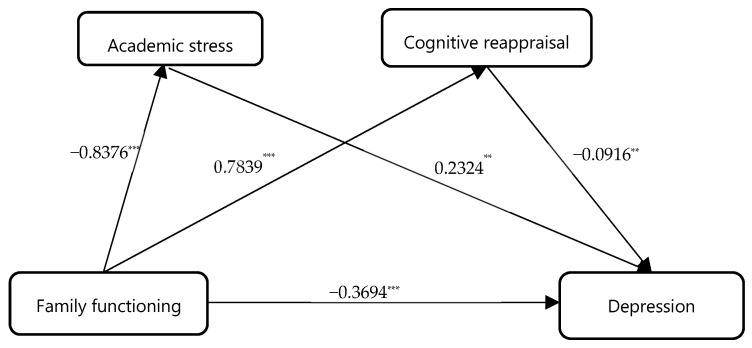
The mediating path diagram of academic stress and cognitive reappraisal. **Note:** *** *p* < 0.001, ** *p* < 0.01.

Since academic stress did not significantly predict cognitive reappraisal, no chain mediation effect was observed in this model. Taken together, these findings indicate that academic stress and cognitive reappraisal operate as parallel mediators in the relationship between family functioning and adolescents’ depressive tendency, functioning through two distinct pathways: family functioning → academic stress → depression, and family functioning → cognitive reappraisal → depression (see Table 5 and Table 6, and Figure 3).

## 4. Discussion

### 4.1. Prevalence and Group Differences in Academic Stress and Depression

Drawing upon questionnaire data from 437 adolescents, the present study explored the prevalence and distribution of academic stress and depressive symptoms. The results indicated that 27.7% of participants met the criteria for depression, with significant differences observed across grade levels. Similarly, 31.2% of students reported experiencing moderate to high levels of academic stress, which also varied significantly by both gender and grade. These findings are largely consistent with previous research (Y. Liu & Lu, 2012), suggesting that academic stress and depression are pervasive concerns among adolescents.

This phenomenon can be interpreted from both external and internal perspectives. Externally, intense competition for academic advancement, prolonged study hours, and frequent examinations expose adolescents to sustained and high-pressure learning environments, potentially triggering chronic stress and anxiety. Internally, adolescence is marked by asynchronous physiological and psychological development, unstable self-identity, limited problem-solving abilities, and recurrent self-doubt. These internal vulnerabilities, intensifying under high parental expectations and critical parenting styles, collectively elevate the risk for academic stress and depression.

Independent samples *t*-tests revealed that female students reported significantly higher levels of academic stress compared to their male counterparts. One plausible explanation is that adolescent girls may be more sensitive to social evaluation and are more likely to equate academic achievement with personal self-worth, leading to increased anxiety when expectations are not fulfilled. In contrast, societal acceptance of boys developing at a slower pace may act as a protective factor, reducing their psychological burden and stress.

Results from a one-way ANOVA demonstrated a significant escalation in academic stress across grade levels. Seventh graders, who are typically undergoing environmental adjustment and acquiring foundational knowledge, reported the lowest stress levels. Stress increased notably among eighth graders, likely due to greater curricular complexity and an expanded academic workload. Ninth graders experienced the highest stress, confronting high school entrance examinations, multiple mock assessments, heightened scrutiny from parents and teachers, greater self-awareness, and increased emotional fluctuation. These converging factors culminated in the most pronounced academic stress among ninth graders, a pattern corroborated by longitudinal evidence (C. Tang et al., 2025).

Although there were no significant gender differences in depression scores, depression did vary significantly across grade levels (*p* < 0.01). With each advancing grade, students faced escalating academic challenges, more frequent examinations, and intensified competition for advancement, all coupled with sharper parental and teacher expectations and increased supervision. This environment contributed to a detrimental cycle of heavy investment, elevated anxiety, and diminished perceived returns, ultimately heightening students’ vulnerability to depressive symptoms.

### 4.2. The Relationship of Family Function, Academic Stress, Emotion Regulation, and Depression

The results demonstrated significant correlations among all measured variables. Notably, family functioning was negatively associated with both academic stress and depression, whereas academic stress was positively correlated with depression. With regard to emotion regulation strategies, cognitive reappraisal was negatively correlated with depression, while expressive suppression exhibited a positive relationship with depression. Collectively, these findings underscore the centrality of family functioning and support the existence of a sequential mechanism in which family factors influence emotional outcomes via their impact on the stress process.

Regression analyses further revealed that the direct effect of family functioning on depression was significant (β = −0.3694, *p* < 0.001), suggesting that family functioning is the most robust predictor of adolescents’ emotional well-being. This finding supports Hypothesis 1 and aligns with prior literature (Yang et al., 2020).

Previous empirical work has similarly indicated that adolescents from well-functioning families are better equipped to mobilize internal resources, such as self-efficacy, to cope with academic stress. Emotional warmth and the provision of autonomy in such families mitigate students’ anxiety regarding academic challenges, thereby reducing the cumulative psychological effects of stress (L. Zhang, 2020). Furthermore, family functioning has been shown to attenuate symptoms of depression and anxiety indirectly by alleviating academic stress (Shi, 2024). This may be attributable to the secure, stable, and nurturing environments created by highly functional families, in which parents offer substantial emotional support, foster positive communication, and actively help their children navigate real-life problems. High-quality parent–child relationships serve to reduce tension and anxiety in academic contexts, thereby promoting a greater likelihood that adolescents will confront challenges with a positive mindset as opposed to succumbing to excessive worry or self-doubt. When confronted with examinations or fluctuations in academic performance, these adolescents are more inclined to employ adaptive coping strategies, which lowers their risk of depressive symptoms.

Academic stress was found to be significantly and positively associated with depression, underscoring its direct detrimental impact on adolescent mental health—a result consistent with previous findings (J. Xu et al., 2010). This relationship may be explained by the fact that academic stress can trigger physiological stress responses and cognitive distortions, which may in turn precipitate academic burnout and intensify negative emotional states such as anxiety and depression, thus perpetuating a vicious cycle of “stress–burnout–emotional deterioration” (D. Li, 2016). For many adolescents, academic achievement is their primary responsibility and main source of self-worth; therefore, academic outcomes exert a substantial influence on psychological well-being. When faced with setbacks, some adolescents internalize the belief that “effort is unrewarded and value is unrecognized,” exacerbating feelings of helplessness and despair. Moreover, academic pressure is often chronic and cumulative, with each subsequent exam and ranking posing a considerable psychological burden. In the absence of effective coping strategies and adequate social support, adolescents are at risk of becoming trapped in chronic stress, which may ultimately progress to depression. Thus, reducing academic burden at its source and enhancing stress-coping capacity represent critical targets for intervention.

Emotion regulation strategies—particularly cognitive reappraisal and expressive suppression—were also found to be closely linked to depression. Cognitive reappraisal was a significant negative predictor of adolescent depression (β = −0.0916, *p* < 0.001), whereas expressive suppression emerged as a positive predictor (β = 0.0764, *p* < 0.001). These findings are consistent with earlier research. For example, J. Wang (2018) identified significant differences in the employment of emotion regulation strategies between depressed and non-depressed individuals: depressed adolescents were more likely to adopt maladaptive strategies, such as expressive suppression, and less likely to utilize adaptive ones like cognitive reappraisal. When confronted with high academic stress, adolescents who habitually engage in cognitive reappraisal tend to experience reduced depressive symptoms, while those who rely on expressive suppression are at greater risk for developing depression. This pattern can be attributed to fundamental differences in the mechanisms underlying each strategy: cognitive reappraisal entails the reinterpretation of stressful events, thereby diminishing the physiological arousal associated with negative emotions and enhancing perceived autonomy; in contrast, expressive suppression involves the inhibition of emotional expression without addressing the underlying distress, leading to the accumulation of unresolved negative emotions and increased psychological burden over time.

### 4.3. Parallel and Serial Mediation Roles of Academic Stress and Emotion Regulation Strategies

This study concurrently investigated the mechanisms through which academic stress and emotion regulation strategies—namely, expressive suppression and cognitive reappraisal—mediate the association between family functioning and depressive tendencies in adolescents.

The results demonstrated that both academic stress and emotion regulation strategies acted as significant mediators in this relationship. Specifically, academic stress and expressive suppression formed a sequential, or chain, mediation pathway from family functioning to adolescent depression, thereby supporting Hypothesis 3. This finding implies that compromised family functioning may amplify adolescents’ perceptions of academic stress, thereby increasing their propensity to employ maladaptive emotion regulation strategies, such as expressive suppression, which ultimately heighten their susceptibility to depression. In this way, these findings contribute to the extension of the contextual model of emotion regulation, clarifying the process by which environmental factors impact psychological health via their influence on stress perception and regulatory behaviors. Notably, these observations are congruent with previous research delineating the mediating role of emotion regulation in the relationship between academic stress and mental health (Cho et al., 2015).

Although the effect size of this chain mediation pathway was modest (indirect effect = −0.0188, accounting for 2.96% of the total effect), its theoretical significance is nonetheless substantial as it reveals an underlying process by which familial environment may contribute to depressive symptoms. In the context of dysfunctional family environments, adolescents may be predisposed to adopt passive and maladaptive internalizing strategies (e.g., suppression or self-blame) when experiencing academic stress, due to apprehension about criticism or a lack of psychologically safe spaces for emotional expression. While defensive tactics such as expressive suppression may provide temporary relief by averting parental conflict or peer rejection, they ultimately deplete cognitive and affective resources, thereby increasing the risk of developing depression over time.

Additionally, the findings indicated that academic stress and cognitive reappraisal functioned as parallel mediators, thus lending support to Hypothesis 2. One plausible reason is that heightened academic stress is more likely to elicit expressive suppression rather than adaptive cognitive reappraisal. Under conditions of elevated stress, cognitive resources are diminished, making it less likely that adolescents will engage in resource-intensive reappraisal and more likely that they will default to automatic, less adaptive suppression strategies. Consequently, academic stress did not serve as a significant predictor for cognitive reappraisal, resulting in two distinct parallel mediation pathways. This suggests that increases in real-world academic stress do not necessarily alter the frequency with which adolescents employ cognitive reappraisal strategies, and that the mechanisms by which academic stress and cognitive reappraisal contribute to depression are largely independent. Academic stress, as an external situational demand, and cognitive reappraisal, as an internal regulatory capacity, appear to operate with a considerable degree of psychological autonomy. Although family functioning exerts influence on both, these effects are parallel and non-interactive rather than sequential or causally interconnected.

Importantly, the mediating effect of academic stress was robust and consistent across both the chain and parallel mediation models—with effect sizes of −0.1892 (29.70% of the total effect) in the chain model and −0.1947 (30.61% of the total effect) in the parallel model. These values were substantially greater than the indirect effects for expressive suppression (effect = −0.069, 10.94%) and cognitive reappraisal (effect = −0.0718, 11.29%). These findings strongly suggest that academic stress represents the most influential mediator in the linkage between family functioning and adolescent depression. Impaired family functioning is thus most likely to intensify perceptions of academic stress and achievement-related anxiety, thereby directly and potently elevating the risk of depression.

Several contextual factors may help explain this pattern. First, academic achievement is often deeply interwoven with family expectations, evaluative feedback, and the overall quality of family relationships, making family dysfunction more readily manifest as academic stress in adolescent experiences. By contrast, emotion regulation strategies such as expressive suppression and cognitive reappraisal are considered more stable individual differences; as such, the effects of family functioning on these strategies tend to be more indirect and their mediating roles weaker. Second, in sociocultural contexts where academic achievement is highly valued, academic performance becomes intimately connected to adolescents’ self-esteem and future aspirations, thus rendering academic stress a persistent and pervasive source of risk for depressive symptomatology. Conversely, while emotion regulation can function as a psychological buffer, its capacity to offset depression may be attenuated in settings characterized by chronic and intense external stressors. Taken together, these findings indicate that, in the context of family functioning and adolescent depression, situational and external pathways—including academic stress—are more salient explanatory mechanisms than individual psychological processes, offering critical insights for the design of targeted interventions.

### 4.4. Intervention Implications

These findings offer clear and actionable directions for family–school collaboration. First, interventions targeting the family system should emphasize strengthening family functioning as a primary preventive strategy. Establishing clear family guidelines, providing consistent emotional support, and nurturing collaborative problem-solving skills can diminish adolescents’ threat perceptions and subsequently lower depression risk. Parents are encouraged to embrace evidence-based educational philosophies, to reject ‘grade supremacy’ mindsets, and to be mindful of the intergenerational transmission of academic anxiety. A crucial shift in parental focus—from academic achievement to children’s emotional well-being, psychological needs, and coping strategies—is essential. This approach entails actively listening to children’s experiences, fostering open dialogue around challenges, and responding to academic fluctuations with empathy rather than criticism. Such practices help cultivate a warm and inclusive family environment, thereby supporting the development of adaptive emotion regulation and psychological resilience.

Second, targeted training in emotion regulation is vital. Given that academic stress readily elicits expressive suppression, school-based interventions should concentrate on identifying and modifying suppression-oriented coping tendencies, while also enhancing cognitive reappraisal skills through cognitive-behavioral approaches. Dedicated courses or group-based programs focused on practical reappraisal (e.g., reframing exams as opportunities for growth and challenging perfectionistic beliefs) can help reduce negative affect (C. Tang et al., 2025). To help students move away from reliance on suppression, schools should educate them about its potential adverse effects (such as emotional numbing, interpersonal alienation, and psychological fatigue), while promoting healthier avenues for emotional expression—including journaling, counseling, and peer support—thereby facilitating a transition from suppression to open communication.

Third, a multi-tiered intervention framework is recommended. For students experiencing heightened academic stress, interventions should center on stress management and the promotion of healthy emotional expression. For those from well-functioning families who nonetheless lack effective coping strategies, targeted cognitive reappraisal training is warranted. At the policy level, homework assignments should be regulated to ensure students retain sufficient time for rest and personal interests, while assessment systems should be revised to emphasize process-based feedback (such as growth portfolios) rather than competitive ranking. During periods of high academic stakes, schools should provide stress-reduction workshops and accessible counseling services to help mitigate anxiety and reduce depressive symptoms.

### 4.5. Limitations and Future Directions

Several limitations should be acknowledged. First, the representativeness of the sample was limited, as it primarily consisted of urban students, with minimal inclusion of rural or special-needs populations. This restricts the generalizability of the findings. Second, reliance on self-report measures introduces the risk of social desirability bias. Future research should supplement self-reports with qualitative methods, such as interviews or diary studies, to more fully capture the dynamic and context-dependent nature of emotion regulation and develop culturally valid measurement tools. Third, the cross-sectional design precludes causal inference, as temporal sequencing cannot be established. For example, depression may increase perceived stress or contribute to deficits in coping strategies. Longitudinal studies are therefore needed to determine causality. Fourth, the omission of potential confounding variables, such as socioeconomic status and parenting style, may have biased the effect estimates.

## 5. Conclusions

This study demonstrates that adolescents commonly experience substantial academic stress and depressive symptoms. Family functioning and academic stress were identified as key predictors of adolescent depression. Notably, academic stress and expressive suppression acted as sequential mediators between family functioning and depressive symptoms, while academic stress and cognitive reappraisal functioned as parallel mediators. Among these mediators, academic stress emerged as the most influential, accounting for approximately 30% of the total effect—significantly more than the combined mediating effects of expressive suppression and cognitive reappraisal (about 10%). Additionally, adolescents exposed to high-pressure environments are more likely to utilize maladaptive emotion regulation strategies, especially expressive suppression.

From a theoretical perspective, these findings underscore the central mediating role of academic stress in the relationship between family functioning and adolescent depression, offering new insight into the mechanisms underlying adolescent depressive symptoms. Practically, several recommendations arise: Parents are encouraged to enhance family functioning, particularly by strengthening communication, emotional support, and problem-solving skills. Managing academic stress should be prioritized as a fundamental component of school-based mental health education. Programs aimed at enhancing adolescents’ cognitive reappraisal abilities—such as cognitive-behavioral therapy or mindfulness-based interventions—should be promoted. Adolescents should be guided to avoid excessive reliance on expressive suppression and adopt more adaptive emotion regulation strategies, particularly in high-stress situations. Furthermore, it is recommended that educational authorities incorporate academic stress assessment into school-based mental health screenings, establish relevant early warning systems, and systematically reduce structural academic pressure.

These conclusions are primarily applicable to adolescent populations in cultural contexts that emphasize academic achievement, where academic stress has distinct socio-psychological consequences. However, as this study utilized cross-sectional data, definitive causal relationships cannot be established. Future research should employ experimental or longitudinal designs to rigorously evaluate the effectiveness of these recommended interventions.

## Figures and Tables

**Table 1:** Differences in Family Functioning, Academic Stress, Emotion Regulation Strategies, and Depression.

		Family Functioning	Academic Stress	Cognitive Reappraisal	Expressive Suppression	Depression
Gender	Male	3.25 ± 0.48	2.06 ± 0.61	5.60 ± 0.96	3.53 ± 1.40	11.09 ± 9.91
	Female	3.13 ± 0.50	2.18 ± 0.70	5.10 ± 1.13	3.77 ± 1.60	12.57 ± 10.12
	t	2.49 *	−1.97 *	4.96 ***	1.71	−1.55
Grade	Grade 7	3.31 ± 0.46	1.99 ± 0.62	5.44 ± 1.07	3.21 ± 1.49	9.56 ± 9.55
	Grade 8	3.14 ± 0.52	2.15 ± 0.72	5.35 ± 1.09	3.78 ± 1.45	12.34 ± 9.87
	Grade 9	3.10 ± 0.48	2.26 ± 0.60	5.30 ± 1.05	4.12 ± 1.47	14.11 ± 10.30
	F	7.828 ***	5.793 ***	0.701	14.253 ***	7.705 ***
		1 > 2 **, 1 > 3 ***	2 > 1 *, 3 > 1 **		3 > 1 ***, 2 > 1 *** 3 > 1 *	2 > 1 *, 3 > 1 ***

**Note:** *** *p* < 0.001, ** *p* < 0.01, * *p* < 0.05; the same below. 1 = Grade 7, 2 = Grade 8, 3 = Grade 9.

**Table 2:** Correlation Matrix of Family Functioning, Academic Stress, Emotion Regulation Strategies, and Depression.

Variables	Family Functioning	Academic Stress	Cognitive Reappraisal	Expressive Suppression	Depression
Family Functioning	1				
Academic Stress	−0.642 **	1			
Cognitive Reappraisal	0.385 **	−0.313 **	1		
Expressive Suppression	−0.395 **	0.334 **	−0.132 *	1	
Depression	−0.642 **	0.606 **	−0.425 **	0.473 **	1

**Note:** ** *p* < 0.01, * *p* < 0.05.

**Table 3:** Regression Analysis of the Mediating Role of Academic Stress and Expressive Suppression (N = 437).

Regression Equation			Fit Index		Coefficient Significance	
Outcome Variable	Predictor Variable	*R*	*R* ^2^	*F*	*β*	*t*
Academic stress	Family functioning	0.6446	0.4155	102.5951 ***	−0.8376	−16.777 ***
	Gender				0.0279	0.5721
	Grade				0.0407	1.3231
Expressive suppression	Family functioning	0.4606	0.2121	29.0768 ***	−0.9104	−5.3063 ***
	Academic stress				0.2932	2.2802 *
	Gender				−0.3751	−2.8724 **
	Grade				0.3213	3.8968 ***
Depression	Family functioning	0.7221	0.5214	93.8982 ***	−0.3584	−7.8349 ***
	Academic stress				0.2259	6.7618 ***
	Expressive suppression				0.0764	6.1506 ***
	Gender				0.0230	0.6758
	Grade				0.0109	0.5039

**Note:** *** *p* < 0.001, ** *p* < 0.01, * *p* < 0.05.

**Table 4:** Mediation Effects of Academic Stress and Expressive Suppression.

Effect	Path	Effect Size	95% CI		Proportion of Total Effect
			Lower	Upper	
Indirect effect	Family Functioning → Academic Stress → Depression(indirect1)	−0.1892	−0.2757	−0.1107	29.70%
	Family Functioning → Expressive Suppression → Depression(indirect2)	−0.0696	−0.1107	−0.0349	10.94%
	Family functioning → Academic Stress → Expressive Suppression → depression(indirect3)	−0.0188	−0.1112	−0.0002	2.96%
Total Indirect effect	indirect1 + indirect2 + indirect3	−0.2776	−0.3683	−0.1928	43.65%
Direct effect	Family Functioning → Depression	−0.3584	−0.4483	−0.2685	56.35%
Total effect	Indirect effect + Direct effect				100%

**Note:** 95% CI denotes the 95% bootstrap confidence interval.

**Table 5:** Regression Analysis of the Mediating Roles of Academic Stress and Cognitive Reappraisal (N = 437).

Regression Equation			Fit Index		Coefficient Significance	
Outcome Variable	Predictor Variable	*R*	*R* ^2^	*F*	*β*	*t*
Cognitive Reappraisal	Family functioning	0.4289	0.1840	32.5450 ***	0.7839	8.1605 ***
	Gender				−0.4084	−4.3499 ***
	Grade				0.0034	0.0571
Academic stress	Family functioning	0.6483	0.4202	78.282 ***	−0.8376	−16.777 ***
	Gender				−0.0279	0.5721
	Grade				0.0407	1.3231
Depression	Family functioning	0.7144	0.5104	89.8679 ***	−0.3694	−7.9964 ***
	Academic stress				0.2324	6.8916 ***
	Cognitive Reappraisal				−0.0916	−5.2286 ***
	Gender				−0.0426	−1.2239
	Grade				0.0364	1.6915

**Note:** *** *p* < 0.001.

**Table 6:** Mediation Effects of Academic Stress and Cognitive Reappraisal.

Effect	Path	Effect Size	95% CI		Proportion of Total Effect
			Lower	Upper	
Indirect effect	Family Functioning → Academic Stress → Depression(indirect1)	−0.1947	−0.2854	−0.1107	30.61%
	Family Functioning → Cognitive Reappraisal → Depression(indirect2)	−0.0718	−0.1134	−0.0346	11.29%
Total Indirect effect	indirect1 + indirect2	−0.2665	−0.3567	−0.1847	41.90%
Direct effect	Family Functioning → Depression	−0.3694	−0.4602	−0.2786	58.10%
Total effect	Indirect effect + Direct effect	−0.6359			100%

**Note:** 95% CI denotes the 95% bootstrap confidence interval.

## Data Availability

The data presented in this study are available on request from the corresponding author. The data are not publicly available due to participant confidentiality and institutional ethical guidelines.
